# Surgery of the Peritoneum

**Published:** 1904-12-31

**Authors:** 


### SURGERY OF THE PERITONEUM.

**Incision and Suture of the Abdominal Wall.**—Stimson[1] recommends a combined transverse and longitudinal incision. The line of the incision is a shallow curve, concavity upwards, and crosses the median line three or four centimètres above the symphysis pubis. The sides extend towards the anterior superior spines, but stop well short of them, the extent depending upon the purpose of the operation and the amount of fat in the abdominal wall. Sometimes a total length of three inches is sufficient. The aponeurosis of the external oblique, having been thus exposed throughout, is divided in the line of the incision, as is also that of the internal oblique, or "sheath of the rectus." The flap is then drawn sharply upwards, and the abdomen opened in the median line in the usual manner. The operation having been completed, the wound is closed by suture of the peritoneum and of the aponeurotic layer posterior to the recti, wherever it can be readily identified and secured, and if the recti tend to fall apart two or three points of suture may be placed in them. The aponeurotic flap is secured in place by chromic catgut sutures. The skin is closed as usual. In no case has any weakness of the abdominal wall manifested itself; the method is easy of execution, ample for any abdominal work that can be done through median incision below the umbilicus, easy of repair, and affords apparently complete protection against post-operative ventral hernia. Dr. Ochsner[2] says there are three elements which must be considered in the closure of abdominal wounds. First, accurate co-aptation; second, an aseptic wound; and third, the absence of pressure necrosis. If these three conditions are carried out it does not make much difference what suture material is used, or what particular method one employs, he will not get post-operative hernia.

**Mesenteric Cysts.**—Baumann[3] quotes Dowd as saying that all forms of mesenteric cysts are divided into three classes: (1) embryonic cysts; (2) hydatid cysts; (3) cases of malignant cystic disease. He therefore shows that with the exception of parasitic cysts and cases of cystic malignant disease all forms of mesenteric cyst are embryonic in origin. Moynihan states that congenital remnants of the Müllerian and Wolffian ducts and bodies and of the vitelline duct are regularly found between the layers of the mesentery. It seems probable that, as Dowd suggests, most mesenteric cysts are derived from the inclusion in the tissues of portions of these structures. A case of dermoid cyst of the mesentery is described by Hall.[4]

**Diffuse Peritonitis** is defined by Blake[5] as an established progressive inflammation without definite limitations. These cases can be subdivided into cases of spreading peritonitis, in which parts of the peritoneum can be demonstrated as free from invasion, and general peritonitis in which no part, possibly excepting the lesser sac, is uninvolved. The methods of treating the exudate in these cases varies. All methods of cleansing the peritoneal cavity are open to objection. He endeavours to minimise as much as possible exposure and traumatism to the intestines. He uses a small incision and irrigates with a large amount of saline solution by means of an irrigating tube, with a central inlet and lateral outlets, which connect with a siphoning rubber tube. This permits a very rapid change of water without increasing materially the intra-abdominal pressure. In the matter of drainage he found it better to drain first through the wound, the drain only entering the peritoneal cavity for half an inch in those cases in which drainage of the peritoneal fossæ is considered unnecessary. The cigarette drain is used and removed in from 36 to 48 hours. This method gives a lessened mortality, a shorter convalescence, more rapid subsidence of temperature, shorter wound-healing, greater freedom from meteorism, and absence of intestinal obstruction. The after-treatment consists in complete rest for the alimentary canal. The stomach is washed out and no food given until the tendency to nausea is past. Nutrient enemata, alternated with saline enemata, are administered if nausea persists. The main point in the treatment of diffuse peritonitis is early diagnosis. The later symptoms of diffuse peritonitis—marked distension, regurgitation, flexed extremities, the facies, and depression from septic absorption—should never be waited for. Brewer points out the danger of evisceration and attempts to remove the exudate, and Eliot considers that the best way to remove the products of inflammation is by lavage with the least possible disturbance of the intestines, and subsequently, as far as possible, in drying the abdominal cavity.[6] A case of pneumococcus peritonitis is recorded by Whipple.[7] The prevention of infection is considered by Mikulicz[8] who, after rejecting the suggestion of a specific serum, and showing the impossibility of disinfecting the mucous membrane of the stomach and intestines preparatory to operation, says that the only resort of the surgeon is to increase the power of resistance against intestinal bacteria by producing an artificial leucocytosis. This condition of hyperleucocytosis has been induced artificially by injections of albumose, decinormal saline solution, bouillon, nucleic acid, and tuberculin. Of these the most satisfactory results have been obtained by means of nucleic acid. A two per cent. solution of nucleic acid is employed, and about 50 c.c. is injected beneath the skin of the chest. As the maximum leucocytosis occurs about 12 hours after injection, Mikulicz regards this period as the proper interval between injection and operation. In most of his cases, in addition to the injections of nucleic acid, the further precaution was taken to flush the peritoneal cavity with warm decinormal saline solution, and it is possible that that contributed, in some measure, to the successful outcome, as saline irrigations, are known to increase the resisting power of the peritoneum.

[1] Annals of Surgery, Aug. 1904, p. 178. [2] Med. Record, May 14, 1904, p. 799. [3] Lancet, May 7, 1904, p. 1272. [4] Ibid., May 14, 1904, p. 1344. [5] Med. Record, Aug. 6, 1904, p. 233. [6] Ibid. [7] Brit. Med. Jour., July 16, 1904, p. 125. [8] Med. Record, July 16, 1904, p. 97, and Lancet, July 2, 1904, p. 1.

